# Skin and gut microbiomes of a wild mammal respond to different environmental cues

**DOI:** 10.1186/s40168-018-0595-0

**Published:** 2018-11-26

**Authors:** Anton Lavrinienko, Eugene Tukalenko, Tapio Mappes, Phillip C. Watts

**Affiliations:** 1 Department of Ecology and Genetics, University of Oulu, 90570 Oulu, Finland; 2 Institute of Biology and Medicine, Taras Shevchenko National University of Kyiv, Kyiv, 03022 Ukraine; 3 Department of Biological and Environmental Science, University of Jyväskylä, 40014 Jyväskylä, Finland

**Keywords:** Anthropogenic impact, Biodiversity, Ionising radiation, Pollution, Skin microbiome, Wild mammal

## Abstract

**Background:**

Animal skin and gut microbiomes are important components of host fitness. However, the processes that shape the microbiomes of wildlife are poorly understood, particularly with regard to exposure to environmental contaminants. We used 16S rRNA amplicon sequencing to quantify how exposure to radionuclides impacts the skin and gut microbiota of a small mammal, the bank vole *Myodes glareolus*, inhabiting areas within and outside the Chernobyl Exclusion Zone (CEZ), Ukraine.

**Results:**

Skin microbiomes of male bank voles were more diverse than females. However, the most pronounced differences in skin microbiomes occurred at a larger spatial scale, with higher alpha diversity in the skin microbiomes of bank voles from areas within the CEZ, whether contaminated by radionuclides or not, than in the skin microbiomes of animals from uncontaminated locations outside the CEZ, near Kyiv. Similarly, irrespective of the level of radionuclide contamination, skin microbiome communities (beta diversity) showed greater similarities within the CEZ, than to the areas near Kyiv. Hence, bank vole skin microbiome communities are structured more by geography than the level of soil radionuclides. This pattern presents a contrast with bank vole gut microbiota, where microbiomes could be strikingly similar among distant (~ 80 km of separation), uncontaminated locations, and where differences in microbiome community structure were associated with the level of radioactivity. We also found that the level of (dis)similarity between the skin and gut microbiome communities from the same individuals was contingent on the potential for exposure to radionuclides.

**Conclusions:**

Bank vole skin and gut microbiomes have distinct responses to similar environmental cues and thus are structured at different spatial scales. Our study shows how exposure to environmental pollution can affect the relationship between a mammalian host’s skin and gut microbial communities, potentially homogenising the microbiomes in habitats affected by pollution.

**Electronic supplementary material:**

The online version of this article (10.1186/s40168-018-0595-0) contains supplementary material, which is available to authorized users.

## Background

Animal skin (SK) and gastrointestinal tracts (GI) host diverse communities of microorganisms (microbiomes) that are important for host health. The community composition of SK and GI microbiomes can impact host fitness via their role in (1) forming a barrier to prevent colonisation by pathogens, (2) helping train the immune system and (3) regulating inflammation [1–3], with the GI microbiota also (4) supplying energy and other important metabolites by fermenting otherwise indigestible foods [4, 5]. It is therefore important to understand the processes that elicit changes in microbiome community composition and the concomitant scale over which individuals exhibit environment-specific microbiomes.

The composition of human SK microbiome may be affected, for example, by geographic location and exposure to certain habitats [6–9], individuality and body site [10], host age and sex [8, 11, 12]. SK microbiomes of animals are less-well studied, but geography, environment and body site [13–17], as well as captivity status [18], determine SK microbiome composition. GI microbiome communities are affected also by factors such as geography, host age [19] and environment [20], but with a clear role for host diet in shaping microbiomes of humans [21, 22] and animals [23–25].

Despite the many studies reporting the important roles of both SK and GI microbiome communities for host health, these communities are typically studied independently of each other. Hence, the potential relationship (or lack of) between the SK and GI microbiomes hosted by an individual has not been established in wildlife (or indeed in domestic or laboratory animals, or humans). Given that individuals’ microbiomes are derived from the environment [26], there is opportunity for a certain level of similarity between the different microbiomes on a host. However, the ultimate composition of, at least, SK and GI microbial communities reflects selection within their distinct niches. For example, skin is mostly dry and aerobic, sparse in nutrients and varies in pH, ultraviolet light exposure, temperature and sebum content [3], while the colon is moist and anaerobic, with a stable temperature and rich in nutrients from host food [27]. Hence, the SK and GI microbiomes in healthy individuals are expected to be dissimilar. As explicit comparisons of GI and SK microbiomes from the same individuals are rare, it is largely unknown whether these microbiomes would converge over the same spatial scale in response to similar environmental cues. Given that the skin and its microbiota have direct contact with the environment [3], it may be predicted that variation in the SK microbiome reflects environmental diversity [6, 9, 16, 28]. In contrast, the GI microbiome is strongly influenced by host diet, whose relationship to the wider environment is not always straightforward [29].

Given widespread human impacts on the environment, it is conceivable that both SK and GI microbiome communities are shaped by the type of human activities [30, 31]. For example, exposure to various chemicals, heavy metals [30] or ionising radiation [32] can change the GI microbiota of laboratory rodents. The impact of most environmental pollutants on SK microbiota is largely unknown, but is predicted to alter SK microbiome diversity and composition [31, 33]. And yet, surprisingly few studies have quantified how anthropogenic habitat modifications affect microbiome composition in wildlife. To the best of our knowledge, the effects of exposure to environmental contaminants on SK microbiota in laboratory or wild mammals have not been studied.

Pollution by radionuclides is a potential source of genotoxicity to humans and wildlife [34, 35], with human activities having led to the release of radionuclides into the environment to leave many persistently contaminated areas worldwide [35]. Notably, the explosion of the reactor four of the Chernobyl Nuclear Power Plant (CNPP) in 1986 released large amounts of radionuclides across some 200,000 km^2^ of Ukraine, Belarus, Russia and parts of Europe [34]. Elevated levels of radionuclides, such as strontium-90 (^90^S), caesium-137 (^137^Cs) and plutonium-239 (^239^Pu), persist around the CNPP, and the adjacent area (~ 30 km radius) has restricted human access in the form of the Chernobyl Exclusion Zone (CEZ). Wildlife inhabiting the CEZ provides the best-studied model of the biological impacts of exposure to environmental radiation [34].

The environment within the CEZ is affected by persistent radionuclide contamination, and the chronic exposure to ionising radiation derived from these contaminants has diverse impacts on living organisms, such as a negative effect on the abundance and diversity of invertebrate assemblages [36–38], bird communities [39, 40] and the density of mammals [41]. Conversely, there is evidence that exposure to elevated levels of radionuclides has little impact on the density of large animals [42]. Similarly, studies on microbial communities have returned conflicting results about effects of radiation on community diversity and abundance. For example, the diversity of free-living microbes inhabiting contaminated areas within the CEZ is apparently reduced [43], similar [44] or more diverse [45] than diversity of microbial communities from control (uncontaminated) areas. Bacterial communities isolated from feathers of birds nesting in areas with elevated (2.9 μSv/h) ambient radiation levels in the CEZ exhibit a reduction in species richness [46]. Exposure to environmental radionuclides within the CEZ altered GI microbiome composition, but not community diversity, in small mammals (bank voles) [47]. No studies have quantified the impact of exposure to environmental radionuclides on SK microbiomes of wild mammals.

Our aim was to (1) quantify the impact of exposure to environmental radiation on SK microbiome of a small mammal, the bank vole *Myodes glareolus*, and (2) to make a direct comparison between the response of SK and GI microbiomes to changes in their environment. To achieve these aims, we analysed samples of fur swabs and faeces collected from animals inhabiting radioactively contaminated areas within the CEZ and from control (no elevated levels of radionuclides) areas within and outside the CEZ. We predicted that bank vole SK microbiomes would be sensitive to radiation exposure, similar to the effect seen in the GI microbiome, and that the SK microbiome would differ at a larger spatial scales, reflecting variation in the environment. We also expect high dissimilarity between SK and GI microbiomes of the same individuals, consistent with their distinct niches.

## Methods

### Bank vole trapping and study design

The bank vole is an ideal mammalian model to study the effects of exposure to radionuclides in the wild, because (1) it is common within and outside the CEZ, (2) bank voles eat contaminated foods and live within and on the soil surface where it can experience considerable (i.e. > 10 mGy/d) absorbed doses of radiation [48]; also, (3) as a small rodent, it has relatively small home range (0.7–0.8 ha) with limited dispersal abilities (up to 1 km in a breeding season) [49], and thus the external radiation exposure of these animals reflects ambient radiation dose rates in their trapping locations. This latter point is important given the mosaic of radionuclide contamination within the CEZ, where contaminated and relatively uncontaminated areas can be separated by about ~ 1.5 km [50].

Bank voles were caught at 70 trapping locations within northern Ukraine (Additional file 1) during May 25–June 19, 2016. At each location, 16 Ugglan Special2 traps (Grahnab, Sweden) were placed in a 4 × 4 grid, with an inter-trap distance of 20 m. The trapping period was up to three consecutive nights in each location, with traps set in the late afternoon and animals collected early in the following morning. Soil radiation levels at all trapping locations were measured at 1 cm above the ground using a Geiger–Mueller dosimeter (Inspector, International Medcom Inc., CA, USA) (Additional file 1).

**Fig. 1:**
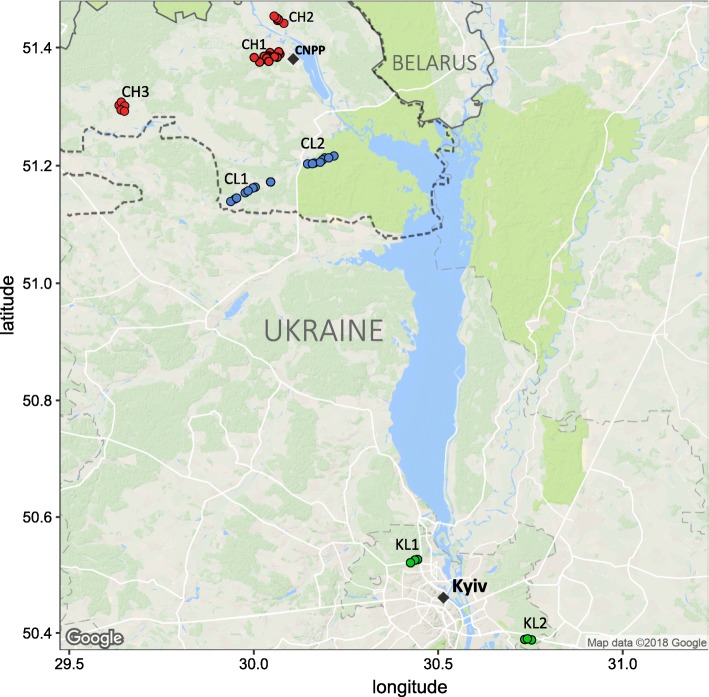
Map of the study areas with bank vole trapping locations shown by points. Replicate sites within each area (e.g. CH1-3, CL1-2 and KL1-2) are shown, with areas contaminated (CH) and uncontaminated (CL) with radionuclides within the Chernobyl Exclusion Zone (CEZ) and uncontaminated area near Kyiv (KL), Ukraine. Colour of the point indicates differences in environmental radiation levels, CH, red (10–198.7 μGy/h); CL, green (0.12–0.55 μGy/h) and KL, green (0.15–0.55 μGy/h). Dashed line represent the border around the CEZ (area of ~ 2050 km^2^). Figure was created using ggmap v.2.6.1 package in R

Our trapping locations represent three general study areas (CH, CL and KL) that contrast in the level of environmental radiation and in their spatial separation (Fig. 1). Within each of these study areas, we sampled two or three replicate (separated by 7–34 km) sites (CH1, 2 and 3, CL1, 2 and KL1, 2) to control for environmental factors other than radionuclide contamination (e.g. potential habitat differences). The three study areas thus represent two environmental radionuclide contamination treatments that differ in ambient radiation dose rates: (1) high (CH, mean = 30.5 μGy/h; range, 10–198.7 μGy/h) and (2) low (CL, mean = 0.26 μGy/h; range, 0.12–0.55 μGy/h, and KL, mean = 0.31 μGy/h; range, 0.15–0.55 μGy/h) radiation. Note, CH sites contained significantly (Bonferroni-corrected Kruskal–Wallis test, *χ*^2^ = 115.4, *df* = 2, *P* < 0.0001) higher levels of radionuclides than did CL and KL sites, with the latter two areas not differing (*χ*^2^ = 115.4, *df* = 2, *P* = 0.718) in level of environmental radioactivity (Additional file 1). As the CEZ is an area of ~ 2050 km^2^ (approximately 30 km radius around the CNPP) and some 80 km from Kyiv (KL), our study areas also examine the effect of spatial separation on SK microbiome: areas within the CEZ (CH and CL) and the areas outside the CEZ (KL).

### Dosimetry

We used individual *γ****-***spectrometry to estimate accumulation of radionuclides (^137^Cs burden) in the whole body of sampled bank voles. Activity of ^137^Cs in bank voles (*n* = 123, representatives of all the study areas) was measured using the SAM 940 radionuclide identifier system (Berkeley Nucleonics Corporation, San Rafael, CA, USA) equipped with a NaI detector (see dosimetry methods details in Additional file 2). Estimated ^137^Cs burden varied from 103.3 to 11,678,418.6 Bq/kg, and animals inhabiting CH on average differed from both CL and KL by more than two orders of magnitude (> 140 times higher) in their ^137^Cs whole-body burden. Thus, voles from CH area had significantly (*χ*^*2*^ = 66.01, *df* = 2, *P* < 0.0001, Bonferroni-corrected Kruskal–Wallis test) higher ^137^Cs burden than did animals from both CL and KL, with the latter two areas not differing in ^137^Cs activity from each other (*P* = 0.227) (Additional file 1).

We also estimated the external radiation exposure of bank voles in our study. External radiation doses varied from 0.10 to 286.13 mGy and averaged around 55.66 mGy in CH and 0.42, 0.45 mGy for CL and KL, respectively (Additional file 1). Similarly as with the ^137^Cs activity, animals from CH experienced significantly (*χ*^*2*^ = 65.96, *df* = 2, *P* < 0.0001, Bonferroni-corrected Kruskal–Wallis test) higher external radiation doses, compared to both CL and KL (not significantly different, *P* = 0.477). Hence, individual-level dosimetry data indicate that bank voles inhabiting CH areas are chronically exposed to significant radiation doses derived from both external (inhabiting the area) and internal sources (for example, from contaminated food).

### Swab sample collection

Bank vole fur was swabbed to sample the SK microbial communities using Sterile Catch-All Sample Collection Swabs (Epicentre Biotechnologies, Madison, USA). Swabs were firmly pressed against the dorsal thoracic area, rubbed back and forth 20 times, and then immediately placed into MoBio Power Bead tubes containing 750 μL of buffer solution (MoBio Laboratories, Carlsbad, USA) and stored at − 80 °C prior to DNA extraction (*n* = 157 total, with CH *n* = 66, CL *n* = 44, and KL *n* = 47 samples). To avoid potential batch effects and systematic bias, samples from each treatment group were processed (e.g. storage, transportation, DNA extraction, library preparation and sequencing) at random. As animals in this study were sampled non-invasively, they were part of the other study (Kivisaari et al. *unpublished*) and were housed in a field laboratory within the CEZ, in individual Makrolon Type III cages (43 × 26 × 15 cm) using sawdust and hey for bedding, with rodent food (RM1, Special Diet Services) and water *ad libitum*. Animal length (nose to anus) and head width was then measured to the nearest 0.1 mm, and weight was measured to the nearest 0.1 g, with gravid females measured after they had given birth. Body mass is often used as a proxy for age in animals, however, body mass is influenced by other factors, for example gravidity status in females. Thus, in this study three age classes were allocated according to head width [51]: juveniles (~ 1 month, 11.6–12 mm) and two age categories of adults (~ 2–5 months, 12.1–13.4 mm and > 10 months, 13.5–14 mm) (Additional file 1). Visual assessment of sex (*n* = 63 males and *n* = 94 females) and maturity at capture was performed relative to animal phenology (i.e. descended testes for males, gravid, lactation or with a perforate vagina for females). Based on the combination of these measurements, very young (~ 1–2 weeks old pups) individuals were excluded and were not sampled for the purpose of the present study.

### DNA extraction and 16S rRNA gene sequencing

DNA was extracted from swab samples (*n* = 157 samples) using a PowerSoil DNA Isolation kit (MoBio Laboratories, Carlsbad, USA) following minor modifications to the manufacturer’s protocol [52]. PCR amplification of 16S ribosomal RNA (rRNA) gene and library preparation was performed by the Institute for Molecular Medicine Finland (FIMM, University of Helsinki) (www.fimm.fi). Briefly, the V4 variable region of the 16S rRNA gene was amplified in a multiplex PCR reaction using the original 515F/806R primer pair [53] and indexes. PCR conditions were as follows: 98 °C for 30 s, followed by 27 cycles at 98 °C for 10 s, at 56 °C for 30 s and at 72 °C for 15 s, and then 72 °C for 10 min. PCR products were pooled in equal volumes and purified twice with an Agencourt AMPure XP PCR Purification kit (Beckman Coulter, Brea, CA, USA) using 0.8× volume of beads compared to library pool volume. The final library was quantified using an Agilent High Sensitivity DNA Kit (Agilent Technologies Inc., Santa Clara, CA, USA) on an Agilent 2100 Bioanalyzer. 16S rRNA gene barcoded amplicons were sequenced on an Illumina MiSeq to provide 250 bp paired-end (PE) reads.

### Read data processing

Sequence data were de-multiplexed by FIMM. Adapter sequences were trimmed in PE mode using Trimmomatic v.0.35 [54]. Overlapping PE reads were assembled using Pear v.0.9.10 [55], with specified minimum (-n 290) and maximum (-m 294) length of assembled sequences. These procedures left 8,012,677 reads that represented an average of 51,036 reads per sample (range = 36–247,393) that were processed using Qiime v.1.9.1 [56]. Potential chimeric sequences were identified and removed using Uchime [57] and the retained (chimera-free) data clustered into operational taxonomic units (OTUs) using SortMeRna and Sumaclust as implemented by Qiime’s open-reference OTU-picking pipeline. Bacterial taxonomy was assigned at 97% sequence similarity against the Greengenes v.13_8 database [58]. Low-abundance (< 0.005% of data) OTUs were removed [59], and the OTU table was rarefied to 10,268 reads/sample (six samples were thus omitted due to insufficient read data) prior to downstream analyses [60]. A core bank vole SK microbiome was defined by the OTUs present in all 151 individuals (and thus all study areas). The final data for SK microbiome analysis represented 1928 OTUs (average of 901 OTUs/sample, range = 562–1200 OTUs/sample) (Additional file 1).

### Statistical analyses

Statistical analyses were performed using R v.3.3.3 [61] unless otherwise stated. Significant differences in alpha diversity (richness—number of observed OTUs; evenness—Shannon index) and community composition among study areas were identified using Kruskal–Wallis tests, followed by a Dunn’s post hoc test using the dunn.test package [62] and a Benjamini–Hochberg false discovery rate (FDR) or a Bonferroni correction for multiple testing. We also completed the Spearman’s rank correlation analysis to examine potential associations between (1) external radiation dose and (2) ^137^Cs whole-body burden and bank vole SK microbiome alpha diversity.

We identified potential predictors of SK microbiome community diversity using generalised linear modelling (GLM). Explanatory variables included in the full model were bank vole sex (male, female), body mass, head width (a proxy for age), study area (CH, CL and KL) and study area by host sex interaction (*). Model selection was based on Akaike Information Criterion (AIC) adjusted for sample size using AICc for model ranking [63]. The most parsimonious model within two AICc units from the model with the lowest AICc was considered as the best model supported by the data. Model selection was carried out using the dredge function in MuMIn v.1.15.6 [64].

Beta diversity was estimated using Bray–Curtis dissimilarities and unweighted UniFrac distances between samples, as calculated by phyloseq v.1.19.1 [65]. Differences among samples were visualised by principal coordinates analysis (PCoA), with statistical significance calculated using the permutation manova (permanova) (999 permutations) implemented by the adonis function in vegan v.2.4-2 [66].

Random forest machine-learning classification was used to determine the accuracy with which bank vole SK microbiomes could be assigned to their own trapping location based on microbiome community composition. Models were run using the randomForest [67] implemented in Qiime v.1.9.1, with 1000 trees per model and 10-fold cross validation error estimate. Models examined a contrast in assignment success (i.e. intra-and inter-group coherence) of bank vole SK microbiomes (1) from within and outside the CEZ, (2) among the three study areas (CH, CL and KL), and (3) among all seven (i.e. replicate) sites.

Our processing of SK microbiome data was identical to our processing of bank vole GI microbiome (Qiita (https://qiita.ucsd.edu/), study ID 11360, EBI accession number ERP104266 [47]). To compare the spatial scale over which the SK and GI microbiomes vary in diversity and community structure, we analysed microbiome data that were available for the same bank voles (*n* = 93 total, with CH *n* = 36, CL *n* = 28, and KL *n* = 29 samples) (Additional file 1). We removed OTUs without a match in the Greengenes v.13_8 database and rarefied both microbiome data sets to an equivalent 9250 reads/sample. Spatial variation in alpha and beta diversity for the SK and GI microbiomes were identified using the analyses described above. We used the Mantel tests (999 permutations) implemented by Qiime v.1.9.1 to examine the strength of any correlation between the geographic distance separating pairs of samples and the associated level of microbiome community dissimilarity (Bray–Curtis and unweighted UniFrac). We completed Mantel tests for SK and GI microbiomes, (1) for all pairs of samples and (2) for pairs of samples within the CEZ only. Finally, we used random forest modelling [67] to contrast the predictive accuracy with which the SK and GI microbiome samples could be assigned to their study area.

## Results

### Bank vole SK microbiome composition

We used 16S rRNA gene sequencing to characterise SK microbiomes of 157 wild caught bank voles from areas that differed in the level of environmental radionuclide contamination (Fig. 1). We identified 1928 unique OTUs from 15 bacterial phyla. Five phyla accounted for ~ 97% abundance: *Proteobacteria* (34%), *Firmicutes* (28%), *Actinobacteria* (16%), *Bacteroidetes* (16%) and *Cyanobacteria* (4%) (Additional files 3 and 4). The class *Gammaproteobacteria* (phylum *Proteobacteria*) predominated (> 20%), followed by *Actinobacteria* (phylum *Actinobacteria*), *Clostridia* and *Bacilli* (phylum *Firmicutes*) that were present at roughly equal abundance (~ 14%). This community composition is comparable with SK microbiomes of other nonhuman mammals [17]. The core bank vole SK microbiome comprised OTUs from the genera *Lactobacillus*, *Arthrobacter*, *Staphylococcus* and *Acinetobacter* and from the order *Streptophyta*. As almost none of the OTUs were restricted to just one study area, variation in abundance of microbial taxa generates the following spatial differences in SK microbiome diversity and community structure.

### Variation in SK microbiome alpha and beta diversity

**Fig. 2:**
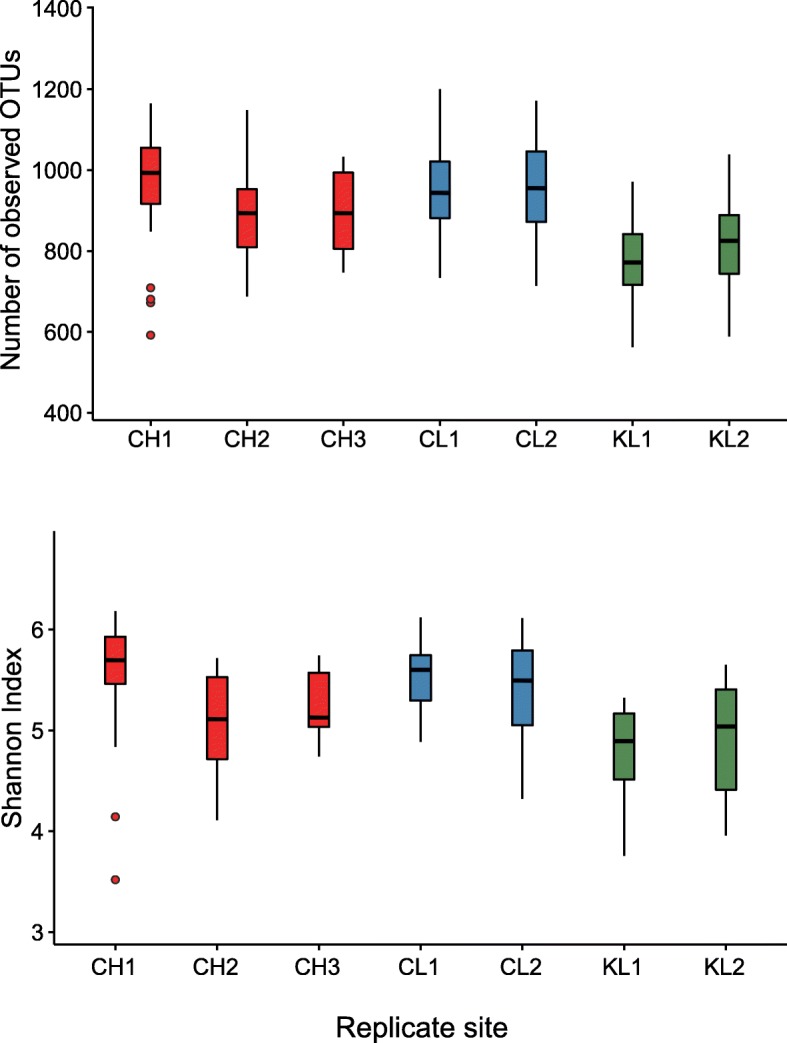
Measures of alpha diversity for the skin microbiome of bank voles inhabiting areas that differ in levels of environmental radiation. Box-and-whisker plots represent the median and interquartile range of alpha diversity estimates (i.e. number of observed OTUs, Shannon index). Each box plot represent a replicate site from contaminated (CH1-3) and uncontaminated (CL1-2) with radionuclides areas within the Chernobyl Exclusion Zone and uncontaminated area near Kyiv (KL1-2), Ukraine

Alpha diversity of the bank vole SK microbiome is affected by geographic location and host sex rather than the level of radionuclide contamination. Neither external radiation dose (*r* = 0.081, *P* = 0.44, Spearman’s correlation analysis) nor whole-body ^137^Cs burden (*r* = 0.11, *P* = 0.29) of sampled bank voles were significantly correlated with SK microbiome alpha diversity (Additional file 5). Alpha diversity of SK microbiome was significantly (*P* < 0.0001, Kruskal–Wallis test) higher in both contaminated (CH) and uncontaminated (CL) areas within the CEZ than outside the CEZ (KL) (Fig. 2 and Additional file 6). We observed substantial variation among replicate sites from the contaminated areas, for example, with SK microbiome from bank voles captured in the Red Forest (one of the most radioactive sites within the CEZ) and adjacent areas having the highest number of unique OTUs (Fig. 2). Neither body mass or head width (a proxy for age) was associated with SK microbiome alpha diversity. Interestingly, male bank voles had significantly (*P* < 0.05, GLMs) greater alpha diversity (number of OTUs and Shannon index) than females, but there was no significant sex by treatment interaction (Additional files 7 and 8). No SK microbial taxa were exclusively found on one sex, and there was no significant difference (*P* > 0.05, Kruskal–Wallis test) in the relative abundance of most of the dominant (top 10) bacterial genera (for example, *Acinetobacter*, *Staphylococcus* and *Pseudomonas*) between the SK microbial communities of male and female bank voles. In contrast, there was marked difference in the relative abundance of bacteria from the *Lactobacillus* genus, with female bank voles having twofold (*P* < 0.0001, Kruskal–Wallis test) more *Lactobacillus* taxa than males. However, among females, variation in *Lactobacillus* did not depend on gravidity status (*P* = 0.16, Kruskal–Wallis test) or number of pups (*r* = 0.13, *P* = 0.23, Spearman’s correlation analysis) in the gravid bank voles.

**Fig. 3:**
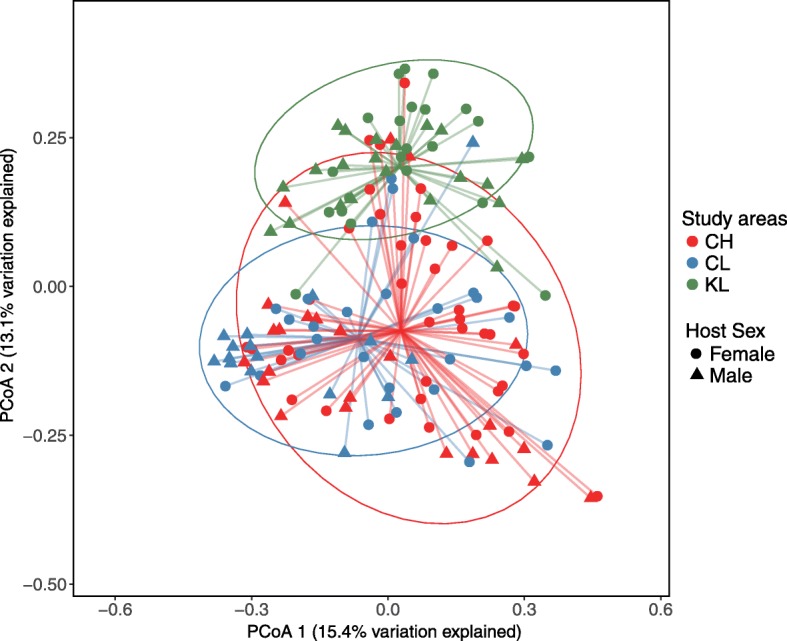
Differences in bank vole skin microbiome beta diversity associated with environmental radiation exposure. PCoA on Bray–Curtis dissimilarity distances between bank vole skin microbiome profiles among the three study areas. Each point represents a single sample (*n* = 151), shape indicate host sex, coloured according to study area: CH, red (*n* = 64); CL, blue (*n* = 44); KL, green (*n* = 43). Ellipses represent a 95% CI around the cluster centroid

Variation in SK microbiome community structure reinforces the pattern of alpha diversity. Bank vole SK microbiome samples from CH and CL characterised by considerably overlapping clusters that were separated from KL samples principally on the second PCoA axis (Fig. 3 and Additional file 9). Hence, bank vole SK microbiome communities significantly differed among study areas (*P* = 0.001, PERMANOVA), with the sample origin from within or outside the CEZ acting as a dominant predictor of SK microbiota beta diversity, based on taxon abundance (Bray–Curtis dissimilarity) and the phylogenetic distance between community members (unweighted UniFrac) (Additional file 10). Notably, radiation was not a significant (*P* > 0.05, PERMANOVA) predictor of the SK microbiome community structure, based on the external radiation dose and whole-body ^137^Cs radionuclide burden (Additional file 10). Consistent with the deliberately heterogeneous sampling, we observed significant (*P* = 0.001, PERMANOVA) differences in beta diversity between replicate sites and this grouping accounted for 13% and 17% of variation in the unweighted UniFrac distances and Bray–Curtis dissimilarity matrices (Additional file 10). Similar to alpha diversity, host sex made a significant (*P* < 0.002, PERMANOVA) contribution to the total variation in beta diversity, with no apparent study area by sex interaction. Bank vole body mass or head width had no statistically significant explanatory effect on SK microbiome community structure (Additional file 10). Hence, the geographical location (within or outside the CEZ) of bank vole trapping sites and host sex are the predominant predictors of bank vole SK microbiome community structure and diversity, with little notable effect of exposure to environmental radionuclides.

### Bank vole origin can be predicted from SK microbiome community composition

That geographic location rather than environmental radionuclide levels affected bank vole SK microbiome composition was further supported by random forest supervised learning. Random forest models correctly classified samples from within (CH and CL) and outside (KL) the CEZ 94% (SD = 5%) of the time; this accuracy is about 4.8-fold greater than expected by chance (Additional file 11), and just one (out of 107) sample from the CEZ was classified as KL. The classifier could correctly assign samples to the three study areas (mean = 80%, ±SD 11%) and seven replicate sites (75 ± 9%), but with a lower (2.8 and 3, respectively) model error ratio (baseline error in case of random guessing to estimated generalisation error of the random forest classifier). This decrease in predictive accuracy arose from the similarity in the CL and CH SK microbiomes within the CEZ; for example, less than 17% of samples from CL1 were correctly assigned, and instead were classified as CH1 (56%) or CH2 (22%). Hence, the high class error for replicates from CL indicate that models were unable to determine whether samples originated from CL or CH within the CEZ (Additional file 11), despite the significant (see the ‘Methods’ section above) differences in the level of external radiation exposure and internal radionuclide burden in animals inhabiting these areas.

### Comparative analysis of the SK and GI microbiomes

**Fig. 4:**
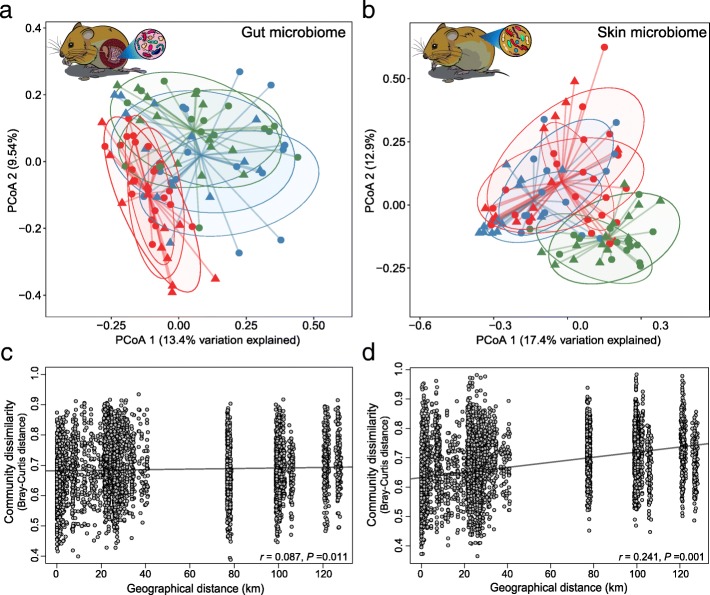
Differences in gut and skin microbiome beta diversity associated with environmental radiation exposure. PCoA on Bray–Curtis dissimilarity distances between bank vole (**a**) gut and (**b**) skin microbiomes profiles among the three study areas. Each point represents a single sample (*n* = 93), shape indicate host sex, coloured according to study area: CH, red (*n* = 36); CL, blue (*n* = 28); KL, green (*n* = 29). Ellipses represent a 75% CI around the cluster centroid for each study area replicate site. Correlation between (**c**) gut and (**d**) skin microbiome communities dissimilarity and geographical distance (km) among bank vole trapping locations. The correlation significance was tested using Mantel tests; lines denote the linear regression model

Bank vole SK and GI microbiomes apparently respond to different environmental cues and thus are structured at different spatial scales. Hence, that geographic location drives significant differences in SK microbiome alpha and beta diversity presents a marked contrast to the GI microbiome where community composition and structure is better defined by the level of radionuclide contamination and not geography (Fig. 4). Thus, while alpha diversity (richness and evenness) of GI microbiome was not significantly (*P* > 0.05, Kruskal–Wallis test) different among the three study areas (and was quantitatively higher in KL), the comparable SK microbiome alpha diversity was significantly (*P* < 0.05, Kruskal–Wallis test) higher within the CEZ (and thus lowest in KL) (Additional files 6 and 12). Moreover, while both SK and GI microbiomes show distinct clustering (*P* < 0.001, PERMANOVA) of samples by study area (Fig. 4a, b), there is a notable overlap in SK microbiome samples from within the CEZ (i.e. samples from CH and CL) whereas the GI microbiome samples cluster according to radionuclide levels (i.e. most overlap occurs between CL and KL, despite the ~ 80 km distance between these locations) [47].

**Fig. 5:**
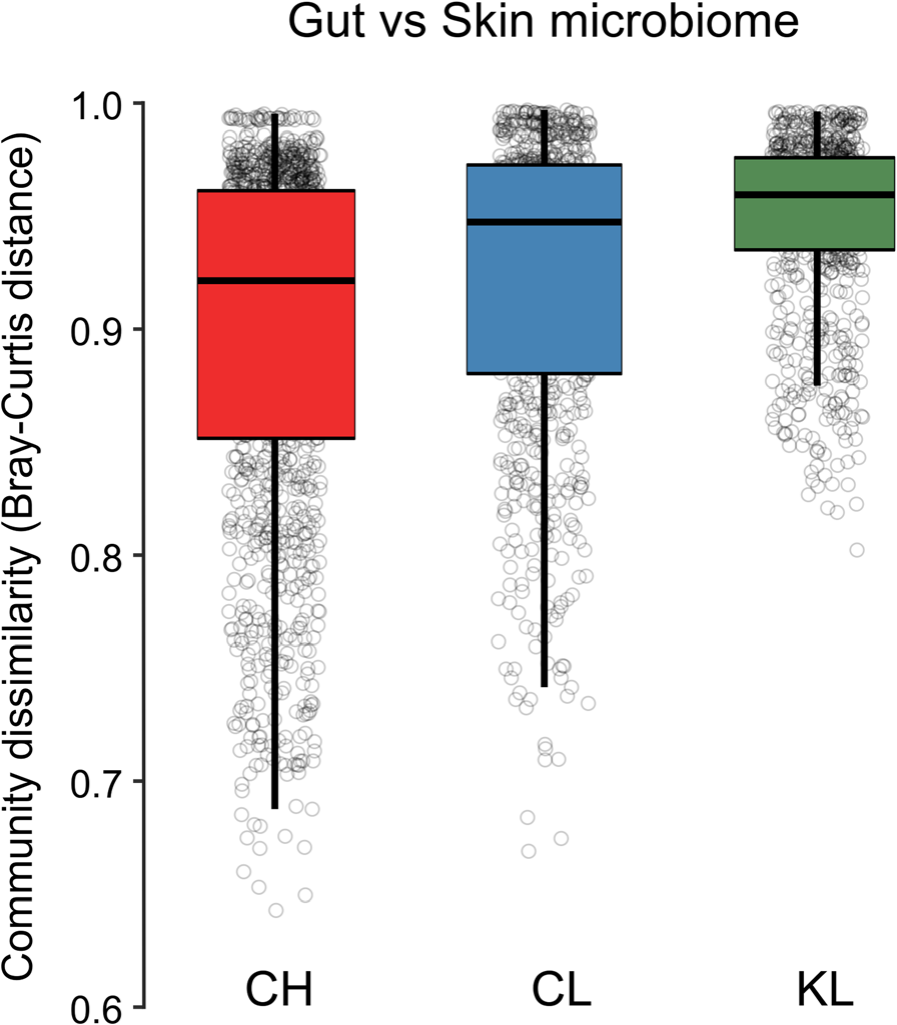
Community dissimilarity between gut and skin microbiomes within each study area. Box-and-whisker plots represent the median and interquartile range of Bray–Curtis distance between samples. Each box plot represent contaminated (CH) and uncontaminated (CL) with radionuclides study areas within the Chernobyl Exclusion Zone and uncontaminated area near Kyiv (KL), Ukraine

**Fig. 6:**
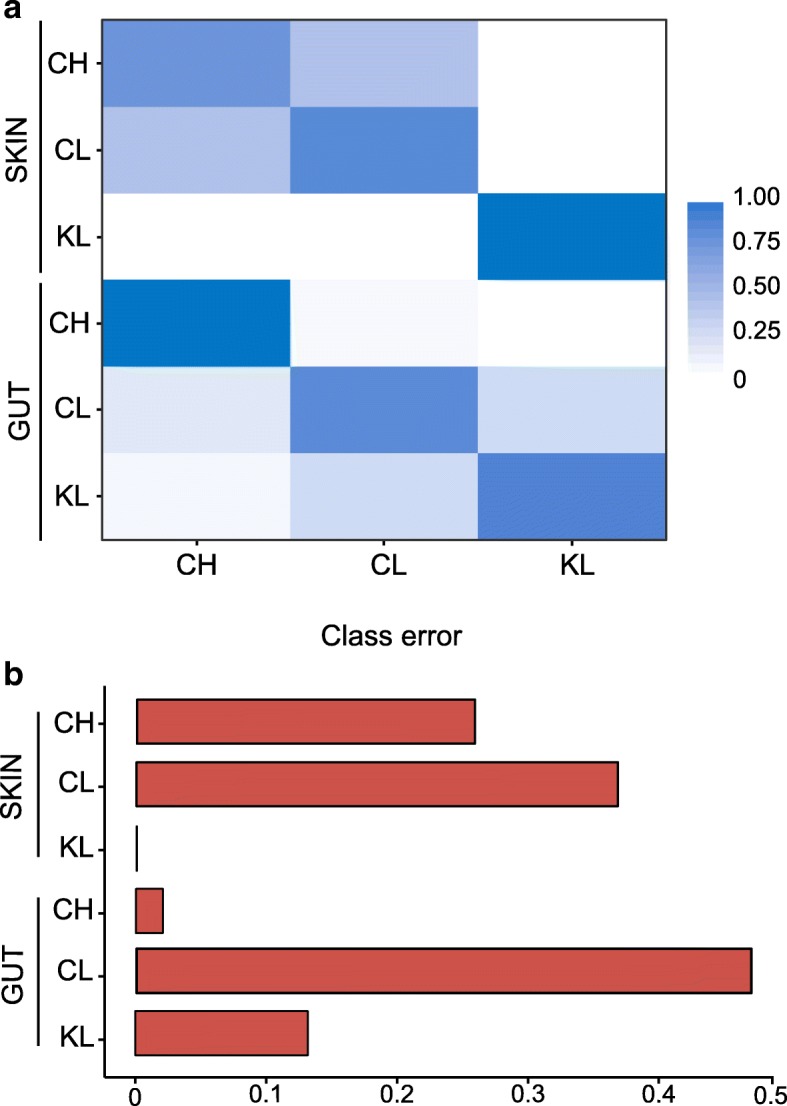
Random forest (RF) classification of skin and gut microbiomes associated with bank voles inhabiting areas contaminated (CH) and uncontaminated (CL) with radionuclides within the Chernobyl Exclusion Zone and uncontaminated area near Kyiv (KL), Ukraine. **a** Each row of the confusion matrix from RF analysis represent the study area, the colour intensity indicates within-group coherence and correspond to the fraction of samples that were predicted by the classifier to belong to the study area specified by each column. **b** Class error estimates indicate the integrity of each study area given the skin and gut microbial communities analysed

Bank vole SK microbiome communities become more dissimilar with increasing geographic distance among bank vole trapping locations (*r* = 0.241, *P* = 0.001 for Bray–Curtis; *r* = 0.222, *P* = 0.001 for unweighted UniFrac, Mantel test, Fig. 4d), further reinforcing the contribution of spatial distance on SK microbiota. In contrast, similarities in GI microbiome communities were weakly, if at all, associated with the distance separating samples (*r* = 0.087, *P* = 0.011 for Bray–Curtis; *r* = 0.016, *P* = 0.706 for unweighted UniFrac, Mantel test, Fig. 4c). Within the CEZ, we identified a significant positive correlation between community dissimilarity and geographic distance for GI microbiome (*r* = 0.163, *P* = 0.001 for Bray–Curtis, Mantel test) only (SK microbiome, *r* = 0.071, *P* = 0.121 for Bray–Curtis, Mantel test). Given that differences in GI microbiome among areas within the CEZ are driven by variation in abundance of bacterial OTUs rather than replacement of taxa [47], it is not surprising that this analysis using unweighted UniFrac as the distance metric was not significant for both SK and GI microbiomes (*r* = 0.072, *P* = 0.155 and *r* = 0.065, *P* = 0.267, respectively, Mantel test). Interestingly, exposure (or potential exposure) to soil radionuclides is associated with a gradient in the level of dissimilarity between SK and GI microbiomes: thus, Bray–Curtis distances were highest (mean = 0.95; range in mean values across KL replicate sites 0.94–0.96) for animals inhabiting locations close to Kyiv (no elevated levels of soil radionuclides), intermediate (mean = 0.92; range in CL replicates 0.91–0.92) at CL locations and lowest (mean = 0.89; range in CH replicates 0.88–0.91) in the animals inhabiting the contaminated (CH) locations (Fig. 5 and Additional file 13). Finally, random forest modelling had similar predictive accuracy for both SK and GI microbiomes (77% and 80%, respectively). However, this analysis was unable to reliably differentiate between samples from CL and CH in SK microbiome, but could not clearly distinguish between samples from CL and KL in case of the GI microbiome (Fig. 6). This further led to constantly high class error estimates for individuals from CL (Fig. 6), which tend to group with CH or with KL, depending on the microbiome (SK or GI, respectively) community analysed (Additional file 11).

## Discussion

Although microbiomes from the skin (SK) and gastrointestinal tract (GI) have diverse implications for host health, the processes that shape community structure and diversity in wild animal microbiomes are poorly understood particularly with regard to anthropogenic habitat impacts. We present the first analysis of the mammalian SK microbiome response to environmental radionuclide contamination, and make an explicit contrast in the scale over which SK and GI microbiome communities differ. Counter to our predictions, exposure to radionuclides has little general impact on SK microbiome of bank voles; rather, bank voles inhabiting the CEZ have higher SK microbiome diversity than bank voles from outside the CEZ. At an individual level, male bank voles have more diverse SK microbiomes. Finally, SK and GI microbiomes respond differently to the environment, with SK microbiome shaped more by geographic location whereas the GI microbiome is affected by local variation in radionuclides levels.

Bank voles have diverse SK microbiota, with the community composition largely similar to that of other non-human mammals [17], and particularly rodents. For example, similar to our data on bank voles, *Gammaproteobacteria*, *Actinobacteria* and *Bacilli* were among the dominant bacterial classes in the SK microbiome of other rodents (squirrel, *Sciurus carolinensis*; groundhog, *Marmota monax*) [17], and also bats [13] and domestic dogs [15]. Within these classes, members from the genera *Acinetobacter*, *Staphylococcus* (notably, *S*. *succinus*), *Lactobacillus* and *Pseudomonas* were associated with bank voles and also the SK microbiota of humans and other mammals [15, 17, 68, 69]; the high prevalence (i.e. found in all samples) and abundance (i.e. within top 10 most abundant genera) of these taxa indicates that they are key members of the bank vole core SK microbiota: determining the functional role of these bacteria in bank vole SK microbiome is an avenue for future research.

### Environment effects on SK microbiome

Most of the SK microbiome studies on non-human mammals have been conducted on captive animals, with just a handful studies examining SK microbiomes of mammals in nature [13] (several bat species), [17] (squirrel, *S. carolinensis*), 18 (Tasmanian devil, *Sarcophilus harrisii*), [70] (humpback whale, *Megaptera novaeangliae*)]. We thus know relatively little about the contributions of environment and host characteristics (for example sex, see below) in shaping the SK microbiome communities in wild mammals. Our results are concordant with the common occurrence of free-living bacteria (for example, soil-associated bacteria from the *Arthrobacter* and *Sphingomonas* genera) within the SK microbiome of non-human mammals [13–15, 17] and on humans from traditional societies [71]. Accordingly, the SK microbiome of a wild rodent is predominantly affected by environmental variation at a relatively large spatial scale (~ 80 km, minimum distance from the bank vole trapping locations within the CEZ and KL), further reinforcing the putative importance of variation in the environment in determining SK microbiome assembly [13, 15, 18, 28].

### Host-specific effects on SK microbiome

Notably, less is known about the impacts of the host characteristics, such as sex, on the SK microbiome community composition of wild animals. In bank voles, variation in head width or body mass (that reflect differences in age or growth, for example) yielded little or no predictive impact on the SK microbiome community diversity, but host sex was important. Sex-biased microbiome community differences are rarely reported, although SK microbiome diversity is higher in human females compared with males [11, 12, 72]. Nonetheless, sex was a significant predictor of the SK microbiota of the red kangaroo (*Macropus rufus*), but not for cats, dogs and horses (that notably are domestic or captive animals) [17]. The twofold increase in bacteria from the *Lactobacillus* genus on female bank vole SK microbiome is consistent with sex differences in humans. Members of the *Lactobacillus* genus dominate the vaginal microbiome [73] and are more abundant in the SK microbiome of females [11, 74]. Hence, it is conceivable that sex differences in SK microbiome community are more general property of the sexes (also due to hormones, secretions, skin thickness) [11, 75]. Sex-specific differences in SK microbiome may also reflect a difference in behaviour that influences the type of environments, and hence microbiota, experienced. For example, as typical of many mammals [76], male bank voles tend to move more to forage and/or find a mate [49]. Greater movements may provide the opportunity for male bank voles to acquire a more diverse microbiota, analogous, for example, to an apparent effect of lifestyle (e.g. time spent indoors or outdoors; inhabiting rural or urban environments; exposure to natural biodiversity) upon animal and human SK microbial diversity [6, 8, 9, 12, 16].

### Effect of exposure to radionuclide contamination

Although anthropogenic habitat modifications have widely reported negative impacts on biodiversity, exposure to environmental radionuclide contamination does not necessarily reduce microbial diversity. This is perhaps curious, as SK microbiomes are derived principally from the environment [13, 15, 28, 71, 77, 78] and processes that negatively affect biodiversity, are expected to have a concomitant effect on SK microbiota [6, 16]. High SK microbiome diversity within the CEZ (compared to KL) thus contrasts with studies on invertebrates [36–38] and microbial communities [43, 44, 46, 47] exposed to Chernobyl fallout, where elevated levels of radionuclides had a negative or no impact on diversity. Similar to our results, soil microbial diversity was higher in a radioactive waste disposal trench in the Red Forest than adjacent sites outside [45]. Forest litter accumulates in contaminated areas (including the Red Forest) within the CEZ [79], potentially increasing available microbial niche space from which bank voles SK microbiome is sourced, given that bank voles inhabit burrows and move about the leaf litter. Curiously, accumulation of forest litter in contaminated areas within the CEZ implies impaired function of microbial decomposers and/or soil invertebrates [79], but see also [80]. As such, high SK microbiome diversity within the CEZ (and by implication high microbial diversity in the surrounding environment) does not appear to be associated with a healthy ecosystem function [81]. Species diversity estimates do not take into account the ecological role and different contributions that species make to the ecosystem services [82].

That the SK microbiomes of animals inhabiting the Red Forest (CH1) and adjacent areas had the highest number of unique OTUs (Fig. 2) might reflect an elevated mutation rate associated with radionuclide exposure [44, 83], although there was no correlation between the SK microbiome alpha diversity and external radiation dose or internal radionuclide (^137^Cs) burden for animals inhabiting these areas (Additional file 5). There is no detailed habitat description of the CEZ that would allow to examine potential role of specific environmental variables on the bank vole SK microbiome assembly. Nonetheless, available historical records suggest that high bacterial diversity in the Red Forest might be a site-specific effect of the habitat remediation, as large amounts of forest debris, vegetation and topsoil from this area were bulldozed into trenches and buried soon after the nuclear accident [45, 84] to potentially create a diverse microbial habitat. Another plausible reason for the ‘elevated’ alpha diversity of SK microbiota in the CEZ is the lack of human actions and thus limited land use (e.g. for forestry, agriculture) in this area. Elevated densities of large mammals in the Belarus sector of the CEZ, for example, are hypothesised to reflect the restricted human access [42]. Both KL replicate sites are located in continuous, largely undisturbed forests. However, as these locations are accessible to humans and relatively close to Kyiv (the 7th most populous city in Europe), there is some potential for anthropogenic impacts on environmental biodiversity, which may lead to an associated reduction in microbial diversity as described for animal and human SK microbiota [6, 8, 12, 16].

### Comparison of SK and GI microbiomes

SK and GI microbiomes have inherent differences in community diversity and apparently respond to different environmental cues. Greater diversity in SK than in GI microbiome of bank voles is consistent with human microbiota [85, 86], but not with other wild mammals [18]. SK and GI microbial communities have important implications for host health, but explicit comparisons of GI and SK microbiomes from the same individuals are rare. While it is reasonable to expect a positive association between microbiome community dissimilarity and spatial separation, this is apparent for the SK, but not GI, microbiome (Figs. 4 and 6). The only other study to compare SK and GI microbiota in a wild mammal (Tasmanian devil, *S. harrisii*) also found a marked effect of geography on the SK microbiota only, while both SK and GI microbiome communities converged in apparent response to their hosts being housed in captivity [18]. Our sampling of replicated contaminated (ca. 7–34 km apart) and uncontaminated areas (separated by ca. 9–80 km) is likely to add noise, but is essential to deconfound potential effects of the radiation exposure (if any) from other environmental factors specific to a certain location [87]. Individual-level dosimetry data, with estimates of bank voles (1) external radiation exposure and (2) whole-body radiation burden (^137^Cs activity), indicate that bank voles sampled from CH were exposed to elevated levels of radiation, but this exposure has little notable effect on the SK microbiota composition. Hence, it is striking that the GI (discussed in details previously, see [47]), but not SK, microbiome respond to locally elevated levels of soil radionuclides (Fig. 4a, b).

Similar to the GI microbiome, the SK microbiome has important dialogue with the host immune system [2, 88]. Host associated microbiota can be commensal, mutualistic or pathogenic, or change their relationship with the host according to the environmental context, such as the inflammatory and metabolic state of the host. While the inflammatory status of sampled bank voles is not known, analysis of the spleen transcriptome (Kesäniemi et al. *unpublished data*) found signs of suppression of the adaptive immune system (impaired antigen processing) in bank voles inhabiting contaminated areas within the CEZ. Environment stress may have implications for host health via changes in microbiomes, for example by transforming some commensal species into opportunistic pathogens and/or inflammatory triggers [89, 90]. Further studies of both GI and SK bacterial functions accompanied with direct measurements of host immune status may help to elucidate mechanistic differences (if any) between the two microbiomes and uncover key members that have implications to bank vole health.

The SK and GI microbiomes in healthy individuals are expected to be different, reflecting selection in their quite distinct niches: the GI tract (colon region) is predominantly composed of dense communities of anaerobic bacteria that facilitate digestion [27] while the skin hosts a diverse, but low biomass of microbiota that tolerates a comparative lack of nutrients [3]. With this in mind, the pattern of increasing similarity between SK and GI microbiomes associated with inhabiting contaminated areas within the CEZ (Fig. 5, and Additional file 13) is striking. It is possible that this reflects greater faecal bacteria in the bank vole SK microbiota. For example, members of the *S24-7* family that almost exclusively found in the guts of homeothermic animals [91] (and which dominate (~ 46%) the bank vole GI microbiota [47]) were also present in the bank vole SK microbiota and were about twofold more abundant in animals from CL and CH (~ 10–12%), compared with KL (~ 4%) (see Additional files 3 and 4). One possible reason for an increase in influence of GI microbiota within the SK microbiome may be altered self-grooming behaviour associated with inhabiting contaminated environments; for example, animals that groom less would ingest less radioactive particles in the contaminated soil. Alternatively, a decrease in self-grooming may indicate a chronic stress response [92, 93]. Given that both SK and GI microbiomes play a major role in health, identifying whether this apparent homogenisation of microbiomes within individuals indicates a dysbiosis associated with radiation exposure requires further study.

Exposure to elevated levels of ionising radiation may impose selective pressure on living organisms, and organisms inhabiting contaminated areas within the CEZ potentially require an appropriate evolutionary response. It is plausible that SK microbiome have adapted to radionuclide exposure, although this explanation fails to account for different spatial scale of community structure exhibited by the SK and GI microbiomes that experience similar radiation exposure within the same bank vole host. It seems unlikely that bacteria from the GI tract are inherently more radiosensitive than SK microbiota, particularly as GI microbiome community differences among sites are driven by variation in abundance of bacterial OTUs rather than replacement of taxa [47]. That said, while the external dose from exposure to soil radionuclides likely affects both SK and GI microbiomes, microbiota inhabiting GI tract may also be subjected to high internal doses of radiation from contaminated foods [94], as internal exposure to radionuclides contribute up to 30% of the total absorbed dose of radiation in bank voles inhabiting the CEZ [94]. Also, as the SK microbiome is determined by contact with the environment, while GI microbiome is largely shaped by host diet [22–25], the discrepancy of GI and SK microbiota may simply be attributed to spatial variation in available diet and environment. For example, within the CEZ the marked change in GI microbiome could reflect the reduced biodiversity of invertebrate communities in the contaminated areas within the CEZ [36–38] that could otherwise form part of the bank vole diet [47]. Indeed, host diet has an immediate impact on GI microbiota [22] and thus, the GI microbiome of bank voles may change and respond to the environment more rapidly. By contrast, the comparative lack of variation in SK microbiomes among bank voles within the CEZ reflects process of continuous acquisition and deposition of environmental microbes by bank voles during movements when foraging and/or to find a mate [49]. Hence, in the absence of a strong effect of radionuclide contamination on taxonomic composition of SK microbiome, divergence in SK microbiota developed at a larger spatial scale, where habitat/environmental differences occur at a scale beyond bank vole dispersal abilities.

## Conclusions

Variation in mammalian SK microbiota is primarily shaped by environmental differences in habitat and host sex, but not by the level of radionuclide contamination. Bank voles inhabiting the CEZ are characterised by high SK microbiome diversity, possibly reflecting a lack of anthropogenic disturbance. This study shows how the SK and GI microbiome communities vary at different spatial scales, presumably reflecting a mismatch in spatial variation in environment and host diet. Finally, we show that inhabiting areas contaminated with radionuclides have the potential to homogenise an individual’s SK and GI microbiomes. Future work will need to examine the adaptive, if any, effects of variation in SK community structure and diversity, as well as the fitness consequences of homogenisation of microbiomes. It is currently unknown whether our findings are specific to bank voles, rodents or represent a more general patter similar across other species. Thus, we suggest that future research should focus on multiple microbial communities across wide range of host species to draw broader ecological and health inferences about environment-host-microbiome interactions.

## Additional files

Additional file 1: 16S rRNA gene sequencing metadata. (XLSX 35 kb)

Additional file 2: Individual dosimetry supplementary information: measurements of the ^137^Cs activity and external radiation dose estimates for sampled bank voles. (DOCX 15 kb)

Additional file 3: Relative abundance (average of taxonomic groups abundance within each sample) of Phyla, Classes, Orders, Families or Genera for skin microbiome of wild-caught bank voles (*Myodes glareolus*) from contaminated (CH) and uncontaminated (CL) areas with radionuclides within the Chernobyl Exclusion Zone and uncontaminated area near Kyiv (KL), Ukraine. (XLSX 91 kb)

Additional file 4: Summary of skin microbiota of the Phyla, Classes, Orders, Families and Genera for wild-caught bank voles (*Myodes glareolus*), with significantly different relative abundances (Kruskal–Wallis tests using Dunn’s post hoc test and followed by a Benjamini-Hochberg False Discovery Rate (FDR) correction) among the study areas (e.g. CL, KL and CH). (XLSX 97 kb)

Additional file 5: Correlations (Spearman’s correlation analysis) between the SK microbiome alpha diversity estimates (Number of observed OTUs and Shannon index) and (**a**, **b**) the whole-body ^137^Cs radionuclide burden, and (**c**, **d**) the external radiation doses of sampled bank voles. All correlations were not significant. (PDF 287 kb)

Additional file 6: Statistical comparison of alpha diversity estimates by Kruskal–Wallis tests using Dunn’s post hoc test and followed by a Benjamini-Hochberg False Discovery Rate (FDR) correction. (XLSX 14 kb)

Additional file 7: Measures of alpha diversity for the skin microbiota of bank voles inhabiting areas that differ in levels of environmental radiation. Box-and-whisker plots represent the median and interquartile range of alpha diversity estimates (i.e. number of observed OTUs, Shannon index). Each box plot represent alpha diversity of the skin microbiome of bank vole females and males from contaminated (CH) and uncontaminated (CL) with radionuclides areas within the Chernobyl Exclusion Zone and uncontaminated area near Kyiv (KL), Ukraine. (PDF 8 kb)

Additional file 8: Final GLMs that show significant predictors of alpha diversity estimates within the skin microbiota of bank voles inhabiting the area within the Chernobyl Exclusion Zone (CEZ) and areas near Kyiv, Ukraine. Only significant models are shown, with significant *P*-values shown in bold. AIC fit criterion is given for each full model. (XLSX 12 kb)

Additional file 9: Differences in bank vole skin microbiome beta diversity associated with environmental radiation exposure. PCoA on unweighted UniFrac distances between bank vole skin microbiome profiles among the three study areas that differ in levels of environmental radioactivity are shown along the first two PC axes. Each point represents a single sample, shape indicate host sex, coloured according to study area: CH, red (*n* = 64); CL, blue (*n* = 44); KL, green (*n* = 43). Ellipses represent a 95% CI around the cluster centroid. (PDF 12 kb)

Additional file 10: Permutational MANOVA (PERMANOVA) statistical tests on unweighted UniFrac (unwUniFrac) distances and Bray-Curtis dissimilarity for skin microbial communities of bank voles inhabiting areas that differ in environmental radiation levels. (XLSX 11 kb)

Additional file 11: Summary of predictive accuracy of Random Forest modelling for skin and gut microbial communities of bank voles inhabiting areas that differ in environmental radiation levels. (XLSX 10 kb)

Additional file 12: Measures of alpha diversity for the skin and gut microbiome of bank voles inhabiting areas that differ in levels of environmental radiation. Box-and-whisker plots represent the median and interquartile range of alpha diversity estimates (i.e. number of observed OTUs, Shannon index). Each point represent a single sample from contaminated (CH) and uncontaminated (CL) with radionuclides areas within the Chernobyl Exclusion Zone and uncontaminated area near Kyiv (KL), Ukraine. (PDF 39 kb)

Additional file 13: Community dissimilarity between gut and skin microbiomes within each replicate site. Box-and-whisker plots represent the median and interquartile range of Bray-Curtis distance between samples. Each box plot represent contaminated (CH1-3) and uncontaminated (CL1-2) with radionuclides study areas within the Chernobyl Exclusion Zone and uncontaminated area near Kyiv (KL1-2), Ukraine. (PDF 43 kb)
