# Supplementary material for: Skin and gut microbiomes of a wild mammal respond to different environmental cues
Source: Microbiome. 2018 Nov 26;6:209. doi: 10.1186/s40168-018-0595-0 (PMC6258405; doi:10.1186/s40168-018-0595-0)
Supplement: Supplementary file 2 — Individual dosimetry supplementary information: measurements of the 137Cs activity and external radiation dose estimates for sampled bank voles. (DOCX 15 kb) [file 40168_2018_595_MOESM2_ESM.docx]

**Additional file 2:**

**Skin and gut microbiomes of a wild mammal respond to different environmental cues**

Anton Lavrinienko^1^*, Eugene Tukalenko^1,2^, Tapio Mappes^3^, Phillip C. Watts^1,3^

^1^Department of Ecology and Genetics, University of Oulu, 90570, Finland

^2^Institute of Biology and Medicine, Taras Shevchenko National University of Kyiv, 03022, Ukraine

^3^Department of Biological and Environmental Science, University of Jyväskylä, 40014, Finland

*Corresponding author: Anton Lavrinienko

E-mail: anton.lavrinienko@oulu.fi

*Dosimetry*

We used individual ***γ-***spectrometry to estimate accumulation of radionuclides (^137^Cs burden) in the whole-body of sampled bank voles. Activity of ^137^Cs in bank voles (*n*=123, representatives of all the study areas) was measured using the SAM 940 radionuclide identifier system (Berkeley Nucleonics Corporation, San Rafael, CA, USA) equipped with a 3"x3" NaI detector. The detector was enclosed in 10 cm thick lead shielding (about 400 kg) to reduce the noise from background radioactivity. The system was calibrated with reference standard sources. After correcting for the laboratory background, the activity of ^137^Cs was assessed from the obtained spectra in the energies window 619-707 keV (with caesium photopeak at 662 keV), with the use of the phantom with known activity and similar to bank vole geometry. Animals were weighted prior to measurements and individual body mass was used to standardize radioactivity across individuals. For each measurement the critical detectable level (decision threshold) was found from Lc=k[Rb/Tb(1+Tb/Ts]1/2, where: Lc – critical level, k – 1,65 (coefficient, which determine 0,05 probability of type I error or false positive), Rb – counting rate of background, Tb – time of background measurement, Ts – time of sample measurement. Activity of ^137^Cs in 25 animals from CL and KL was below Lc (detectable level), when system was not able to reliably distinguish between the read measurement of a sample and background radiation levels (Additional file 1). The initial ^137^Cs activity was regressed to the trapping time point with the model y=ae^λx^, where: y – calculated initial activity of caesium, a – 137Cs activity that was measured in time x hours after trapping, e – 2,72, λ – 0,0139 (caesium elimination constant), x – hours after trapping . The ^137^Cs excretion parameters for the bank vole (elimination constant, time of ^137^Cs half-life) was estimated from the multiple repeated γ-spectrometry measurements (Tukalenko et al. *unpublished*). Estimated ^137^Cs burden varied from 103.3 to 11,678,418.6 Bq/kg, and animals inhabiting CH on average differed from both CL and KL by more than two orders of magnitude (>140 times higher) in their ^137^Cs whole-body burden. Thus, voles from CH area had significantly (Bonferroni-corrected Kruskal–Wallis test, *χ^2^*=66.01, *df*=2, *P*<0.0001) higher ^137^Cs burden than did animals from both CL and KL, with the latter two areas not differing in ^137^Cs activity from each other (*P*=0.227) (Additional file 1).

External radiation exposure of bank voles correlated to the ‘residential’ ambient dose rates, thus dose estimates for individual bank voles derive from the ambient radiation dose rate in their trapping locations (in µGy/h), multiplied by the individual age (time spend in the area) (Additional file 1). External radiation doses of bank voles in our study varied from 0.10 to 286.13 mGy, and averaged around 55.66 mGy in CH and 0.42, 0.45 mGy for CL and KL, respectively (Additional file 1). Similarly as with the ^137^Cs activity, animals from CH experienced significantly (*χ^2^*=65.96, *df*=2, *P*<0.0001) higher external radiation doses, compared to both CL and KL (not significantly different, *P*=0.477). Notably, the direct ***γ-***spectrometry data of the ^137^Cs burden in bank voles was strongly positively correlated (*r*=0.76, *P*<2.2x10^-16^, Spearman’s correlation analysis) to the external radiation dose estimates. Hence, individual-level dosimetry data indicate that bank voles inhabiting CH areas chronically exposed to significant radiation doses, derived from both external (inhabiting the area) and internal sources (for example, from contaminated food).
