# Supplementary figures and images for: Skin and gut microbiomes of a wild mammal respond to different environmental cues
Source: Microbiome. 2018 Nov 26;6:209. doi: 10.1186/s40168-018-0595-0 (PMC6258405; doi:10.1186/s40168-018-0595-0)

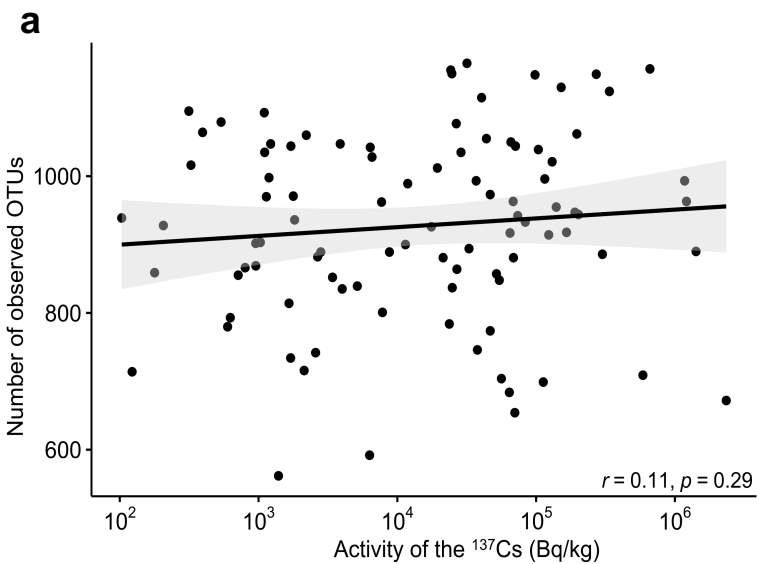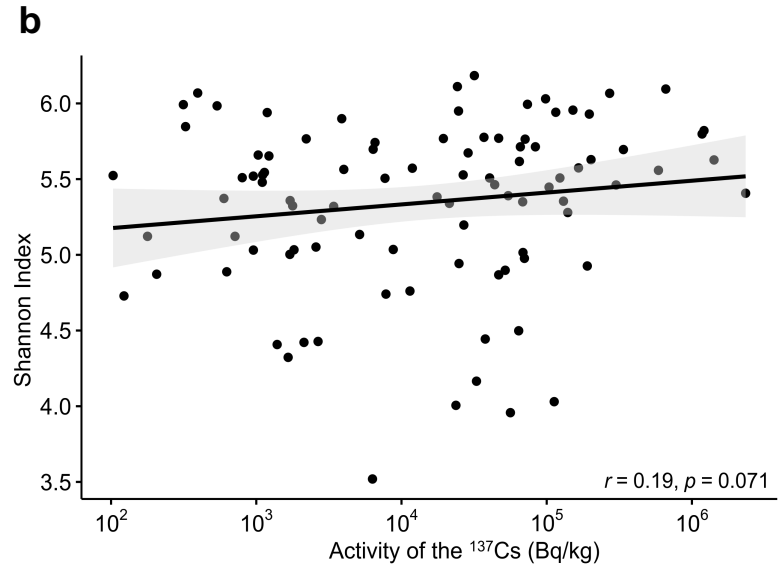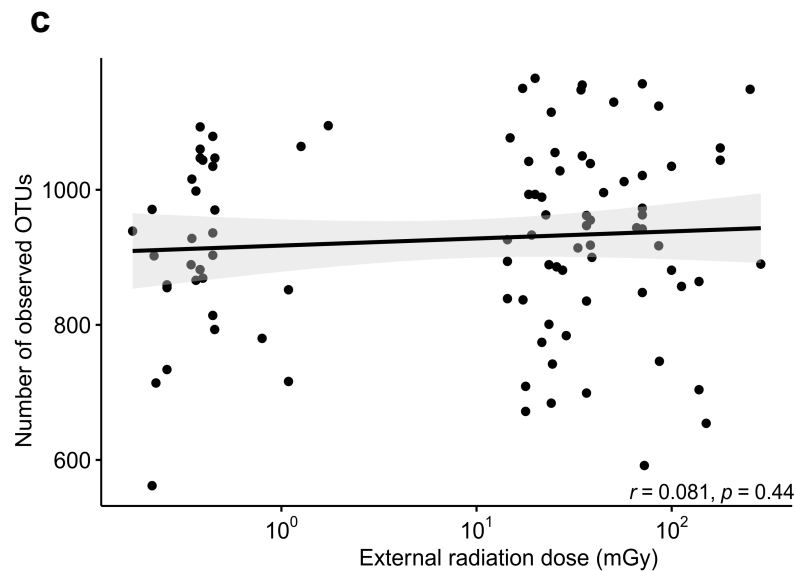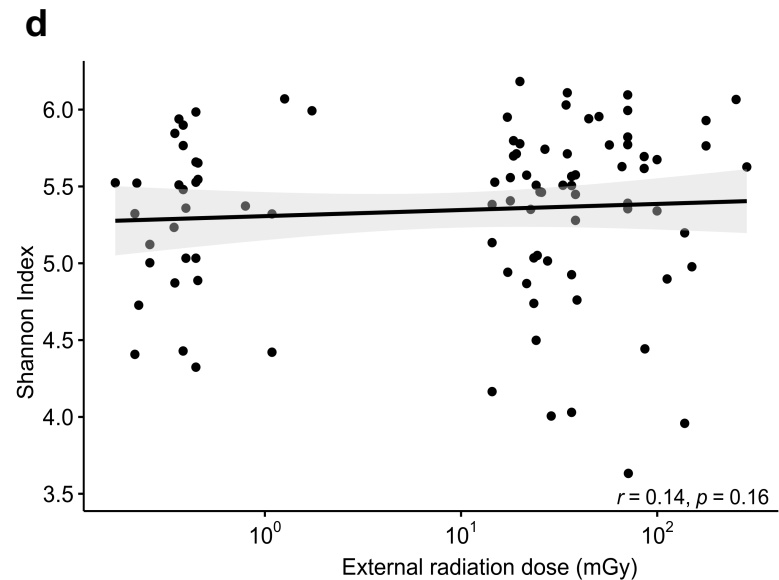

Supplement: Supplementary file 5 — Correlations (Spearman’s correlation analysis) between the SK microbiome alpha diversity estimates (Number of observed OTUs and Shannon index) and (a, b) the whole-body 137Cs radionuclide burden, and (c, d) the external radiation doses of sampled bank voles. All correlations were not significant. (PDF 287 kb) [file 40168_2018_595_MOESM5_ESM.pdf]

# Bank vole skin microbiome alpha diversity

Host sex Female Male

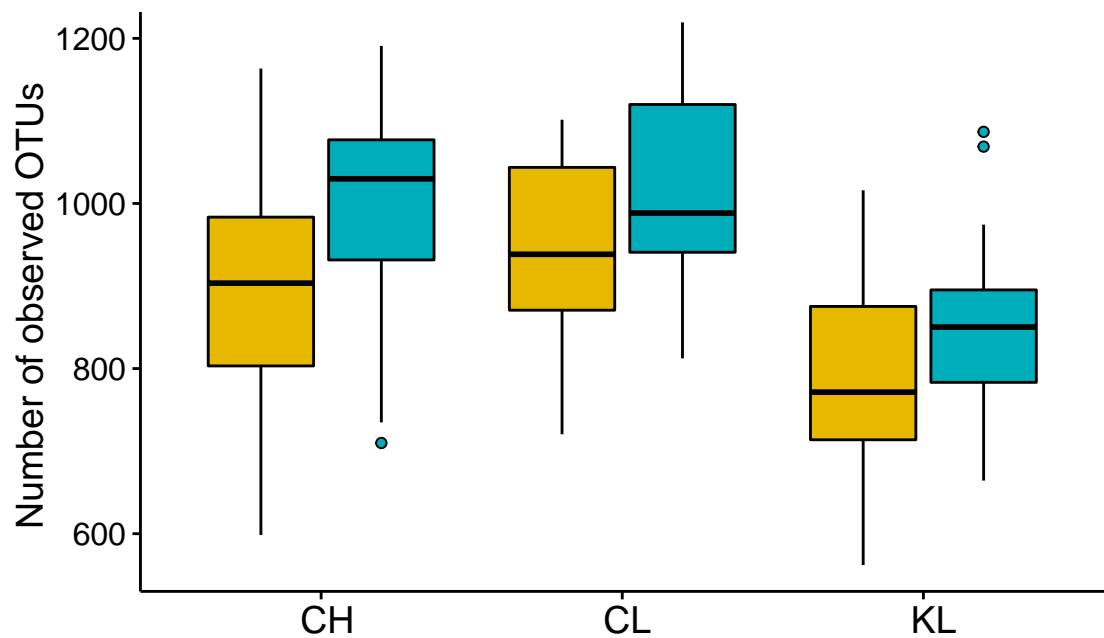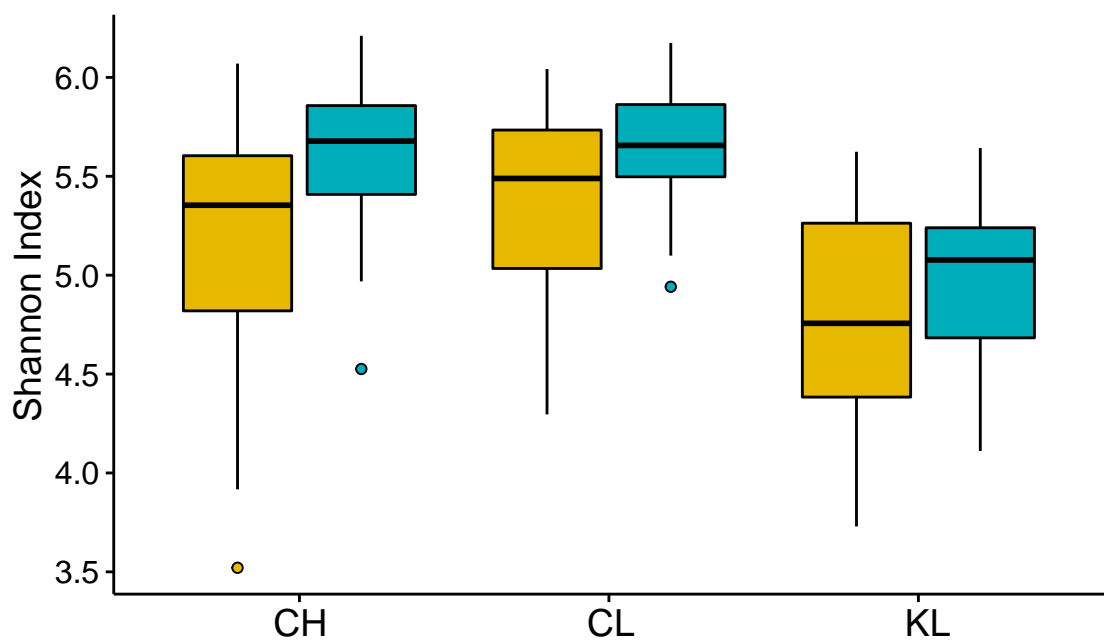

Supplement: Supplementary file 7 — Measures of alpha diversity for the skin microbiota of bank voles inhabiting areas that differ in levels of environmental radiation. Box-and-whisker plots represent the median and interquartile range of alpha diversity estimates (i.e. number of observed OTUs, Shannon index). Each box plot represent alpha diversity of the skin microbiome of bank vole females and males from contaminated (CH) and uncontaminated (CL) with radionuclides areas within the Chernobyl Exclusion Zone and uncontaminated area near Kyiv (KL), Ukraine. (PDF 8 kb) [file 40168_2018_595_MOESM7_ESM.pdf]

PCoA 2 (7.17% variation explained)

0.2  
0.1  
0.0  
-0.1  
-0.2

-0.25

0.00

0.25

0.50

PCoA 1 (18.9% variation explained)

Study area

● CH

● CL

● KL

Host Sex

● Female

▲ Male

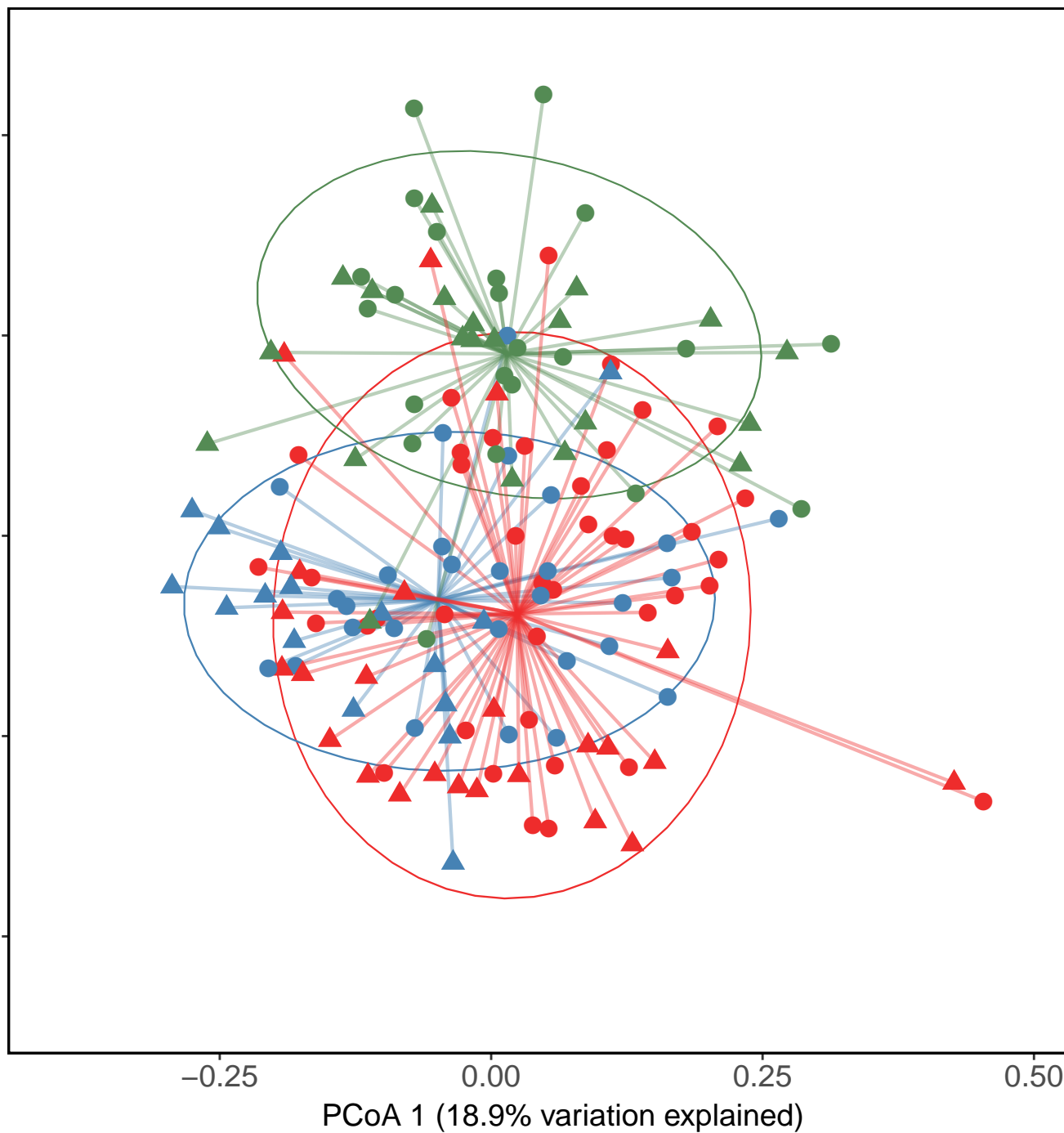

Supplement: Supplementary file 9 — Differences in bank vole skin microbiome beta diversity associated with environmental radiation exposure. PCoA on unweighted UniFrac distances between bank vole skin microbiome profiles among the three study areas that differ in levels of environmental radioactivity are shown along the first two PC axes. Each point represents a single sample, shape indicate host sex, coloured according to study area: CH, red (n = 64); CL, blue (n = 44); KL, green (n = 43). Ellipses represent a 95% CI around the cluster centroid. (PDF 12 kb) [file 40168_2018_595_MOESM9_ESM.pdf]

# Number of observed OTUs

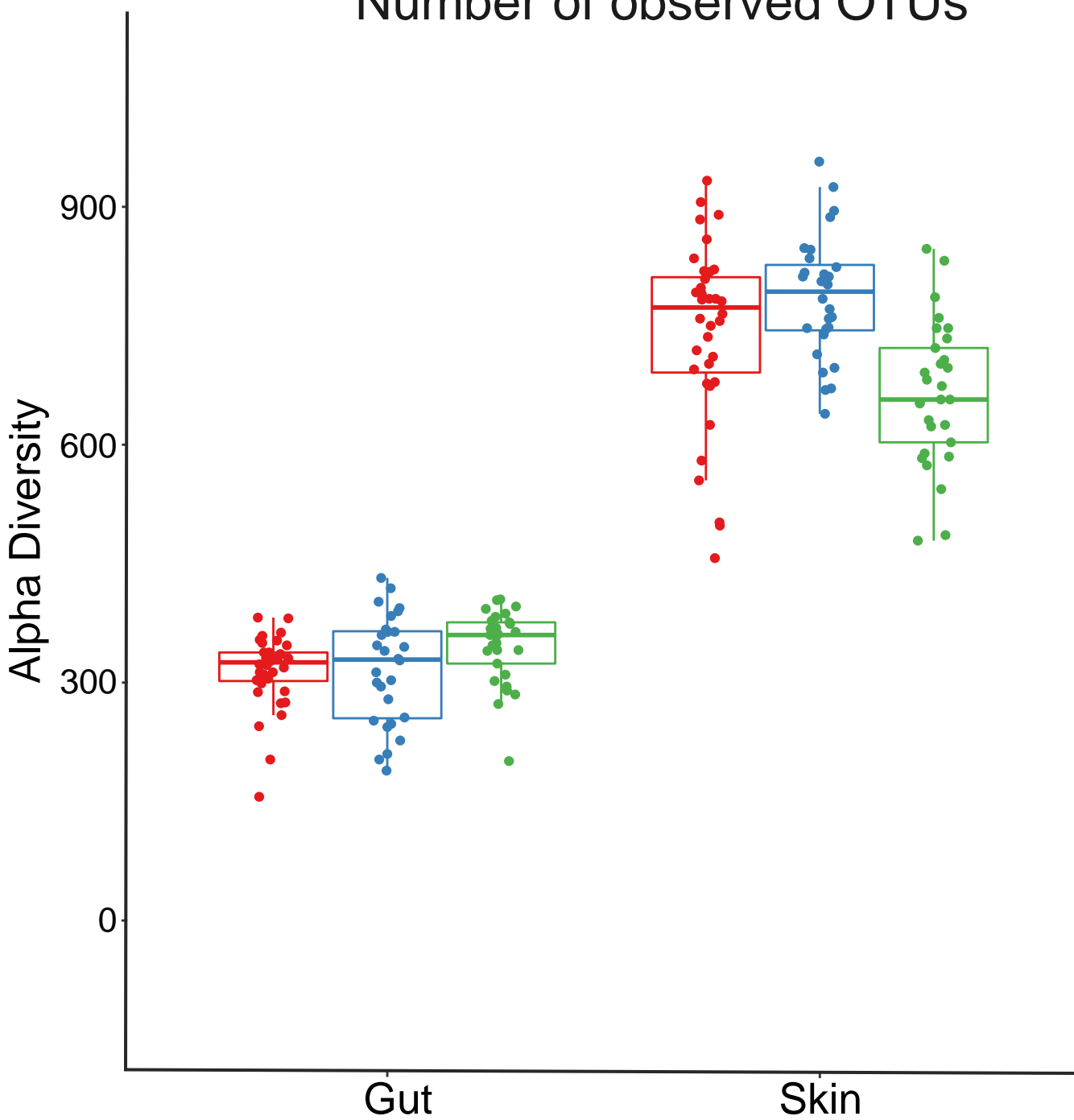

# Shannon Index

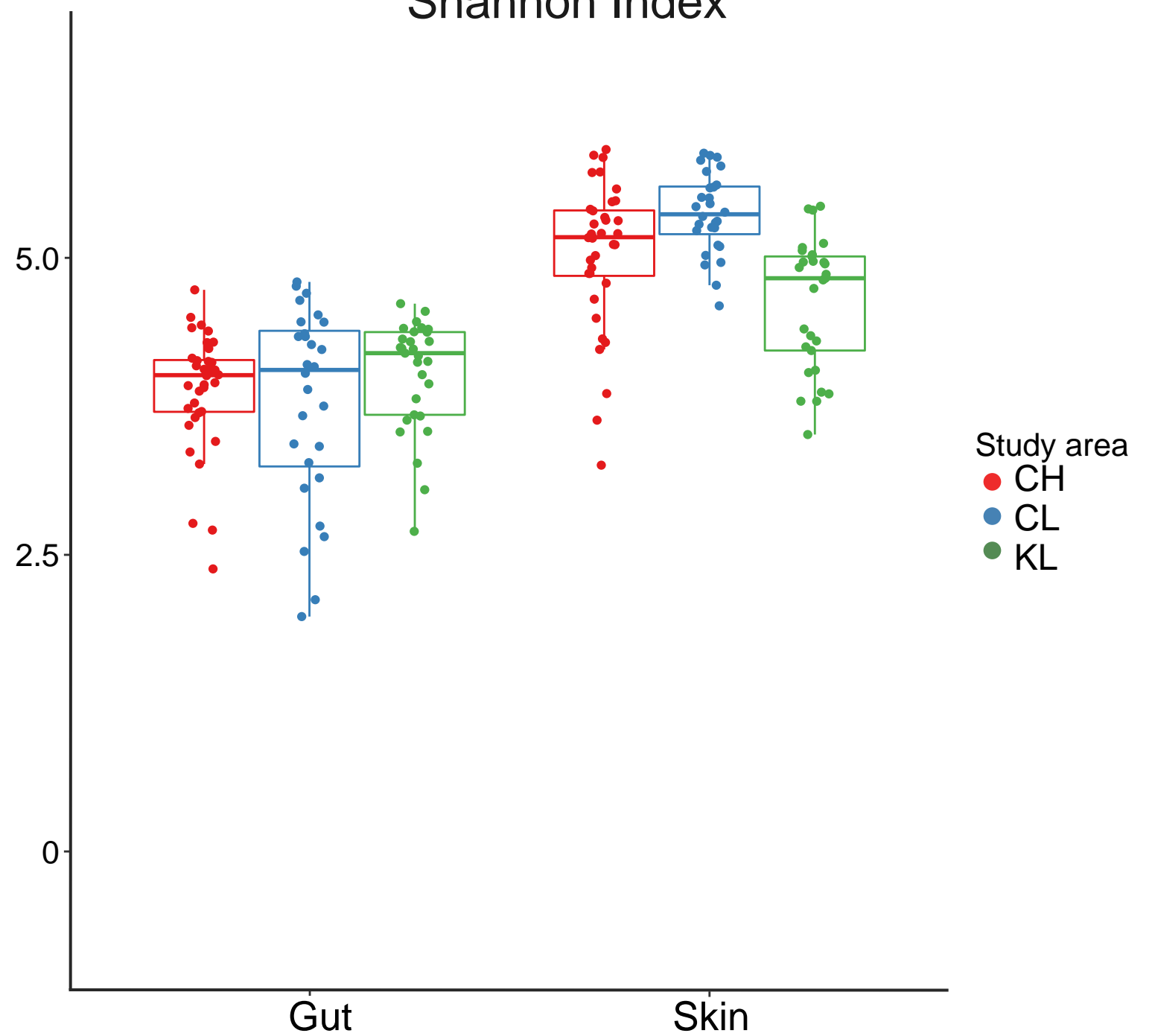

Supplement: Supplementary file 12 — Measures of alpha diversity for the skin and gut microbiome of bank voles inhabiting areas that differ in levels of environmental radiation. Box-and-whisker plots represent the median and interquartile range of alpha diversity estimates (i.e. number of observed OTUs, Shannon index). Each point represent a single sample from contaminated (CH) and uncontaminated (CL) with radionuclides areas within the Chernobyl Exclusion Zone and uncontaminated area near Kyiv (KL), Ukraine. (PDF 39 kb) [file 40168_2018_595_MOESM12_ESM.pdf]

# Gut vs Skin microbiome

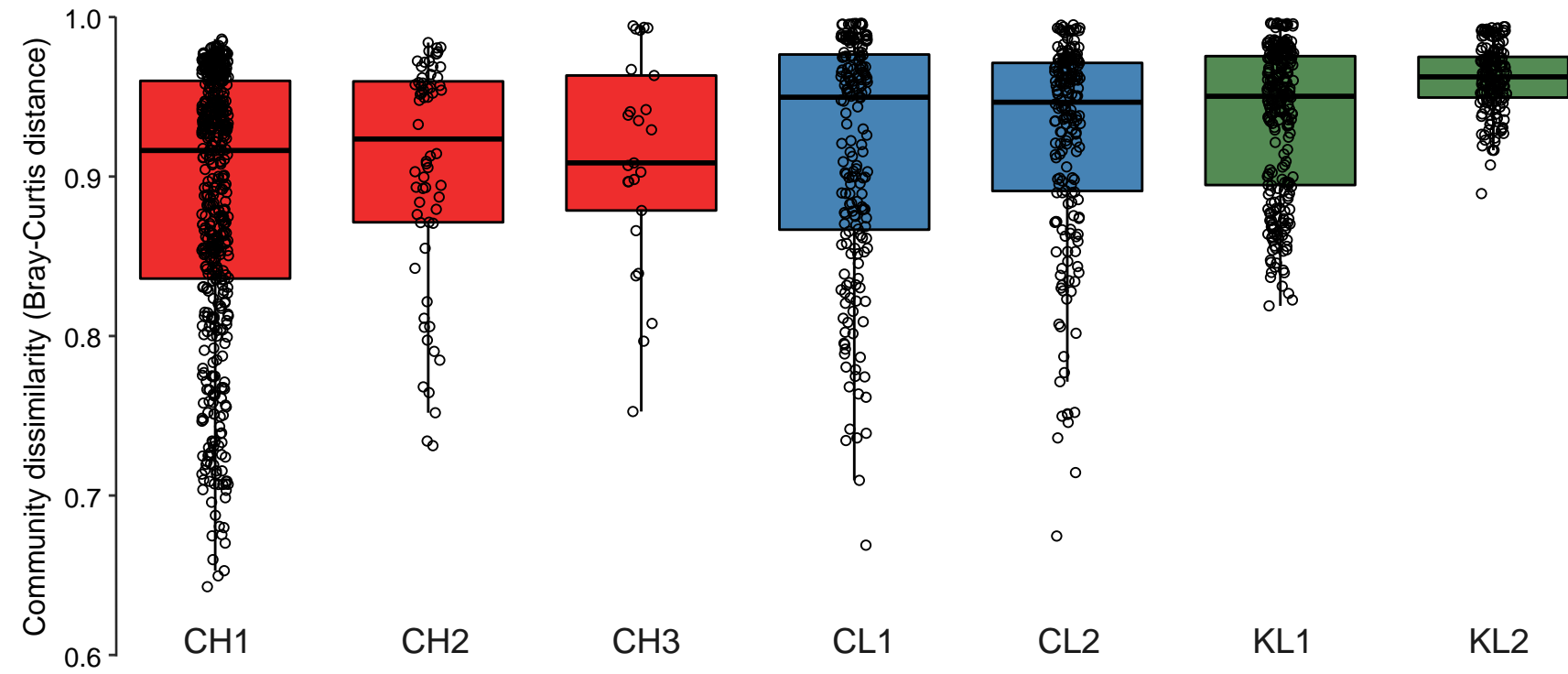

Supplement: Supplementary file 13 — Community dissimilarity between gut and skin microbiomes within each replicate site. Box-and-whisker plots represent the median and interquartile range of Bray-Curtis distance between samples. Each box plot represent contaminated (CH1-3) and uncontaminated (CL1-2) with radionuclides study areas within the Chernobyl Exclusion Zone and uncontaminated area near Kyiv (KL1-2), Ukraine. (PDF 43 kb) [file 40168_2018_595_MOESM13_ESM.pdf]
